# Hard Decision-Based Cooperative Localization for Wireless Sensor Networks

**DOI:** 10.3390/s19214665

**Published:** 2019-10-27

**Authors:** Zhaoyang Wang, Xuebo Jin, Xiaoyi Wang, Jiping Xu, Yuting Bai

**Affiliations:** 1 School of Computer and Information Engineering, Beijing Technology and Business University, Beijing 100048, China; wangzhaoyang@bit.edu.cn (Z.W.); jinxuebo@btbu.edu.cn (X.J.); xujp@th.btbu.edu.cn (J.X.); baiyuting@btbu.edu.cn (Y.B.); 2 School of Automation, Beijing Institute of Technology, Beijing 100081, China

**Keywords:** hard decision, WSN, cooperative localization, distance calibration, outlier constraints

## Abstract

Reliable and accurate localization of objects is essential for many applications in wireless networks. Especially for large-scale wireless sensor networks (WSNs), both low cost and high accuracy are targets of the localization technology. However, some range-free methods cannot be combined with a cooperative method, because these range-free methods are characterized by low accuracy of distance estimation. To solve this problem, we propose a hard decision-based cooperative localization method. For distance estimation, an exponential distance calibration formula is derived to estimate distance. In the cooperative phase, the cooperative method is optimized by outlier constraints from neighboring anchors. Simulations are conducted to verify the effectiveness of the proposed method. The results show that localization accuracy is improved in different scenarios, while high node density or anchor density contributes to the localization. For large-scale WSNs, the hard decision-based cooperative localization is proved to be effective.

## 1. Introduction

Location awareness is rapidly becoming an essential feature of many commercial, public service, and military wireless networks [1,2,3]. Most of the time, data recorded from a wireless sensor only make sense if correlated with a position. For example, the sensors in the wireless sensor network (WSN) for environment monitoring need to be accurately oriented or localized in order to sense, report, and process relevant environmental events [4,5]. Conventional techniques are not adequate for providing seamless and high-accuracy location awareness in harsh environments. For example, the global positioning system (GPS) does not operate well indoors or in urban canyons due to signal blockage [6,7,8]. Hence, new localization techniques are required to meet the increasing need for accurate localization in such harsh environments.

Inspired by their promising applications, researchers have developed many localization systems using different wireless measurements, which are categorized as range-based and range-free methods. The range-based methods, such as time of arrival (TOA) [9], time difference of arrival (TDOA) [10], angle of arrival (AOA) [11,12], and received signal strength index (RSSI), assume that a node is able to measure the distances or angles with respect to its neighbors. The range-free methods, such as Distance Vector-Hop (DV-HOP) [13], Approximate Point-in-Triangulation (APIT) [14], Centroid [15], and Localization Algorithm using Expected Hop Progress (LAEP) [16], assume that a node has no ability to measure the distances or angles with respect to its neighbors and uses connectivity or hop-count information to estimate the positions of the nodes. The range-based localization provides higher accuracy for distance measurement, but extra hardware required by the nodes increases its cost. The range-free localization has a reduced cost, but its accuracy is also relatively decreased. In this paper, we explore a localization technology in large-scale networks characterized by a large node amount and a large node distribution area. Considering the cost requirement for large-scale WSNs, range-free methods are mainly considered in this paper.

Compared with range-based methods, the reason for the lower localization accuracy of range-free methods is the inaccurate estimation of node distance. To improve the localization accuracy, most research in the field is focused on the improvement of distance accuracy. Multi-hop distance estimation is one of the most popular ways to improve distance accuracy. In the field of multi-hop distance estimation, Ta et al. [17] provided an analytical recursive equation for the probability that any two sensors separated by a known distance *x* are k-hop neighbors for any positive integer *k*. Wang et al. [18] derived the expected hop progress from a network model for WSNs in terms of network parameters. Since distance estimation is a key issue in localization systems for WSNs, the proposed range-free LAEP achieves better performance and less communication overhead compared to other existing schemes. Vural et al. [19] proposed a greedy method of distance maximization and evaluated the distribution of the obtained multi-hop distance through analytical approximations and simulations. Zaidi et al. [20,21] considered that the per hop length (PHL) between different nodes might be greatly different in anisotropic WSNs, resulting in a large error in distance estimation. They proposed a novel range-free localization algorithm and derived its average location estimation error in closed form. Wang et al. [22] proposed an error compensation-based node distance estimation, which benefits from joint exploitation, at no cost, of the information provided by the adjacent areas and nodes. By exploring new ways to improve distance estimation, multi-hop methods have been applied to network localization with success. However, the information from anchors has not been fully used, since their characteristics do not accord statistical relationship.

Apart from node distance, the positions of the anchors are also essential to estimate a node position. Typical localization systems for wireless networks employ two types of nodes, i.e., anchors (infrastructures with known positions) and agents (devices with unknown positions). As the anchors and agents play different roles, the existing localization methods can be grouped in two broad categories that may be referred to as non-cooperative localization and cooperative localization. In a non-cooperative localization network, the agents do not receive the position information from other agents. Therefore, the working status of the agents will not affect the localization result. The related methods have been studied in [23,24,25,26,27]. In a cooperative localization network, the agents can obtain distance measurements from their neighboring agents. The localization method considers not only the measured positions of the anchors but also the virtual positions of the agents. Because the agent information is fully utilized in cooperative localization, its performance can be improved.

In the past decade, a plethora of cooperative localization algorithms based on different position-related signal metrics has been proposed. Wymeersch et al. [28] gave an overview of cooperative localization approaches and applied them to ultrawide bandwidth (UWB) wireless networks. They also presented a powerful localization algorithm by mapping a graphical model for statistical inference onto the network topology, which resulted in a net-factor graph, and by developing a suitable net-message passing schedule. Shen et al. [29] established the fundamental limits of wideband cooperative location-aware networks and provided a geometrical interpretation of equivalent Fisher information (EFI) for cooperative networks. This approach helps succinctly derive fundamental performance limits and their scaling behaviors and to treat anchors and agents in a unified way from the perspective of localization accuracy. Chen et al. [30] proposed a new type of power management strategies where each agent individually minimizes its square position error bound penalized by its power cost. The strategies operate as solutions to two power management games that are formulated knowing local information and global information, respectively. Cooperative localization is an emerging paradigm that circumvents the need for high-power, high-density anchor deployment and offers additional localization accuracy by enabling the agents to help each other in estimating their positions. However, most cooperative localization methods only apply to range-based situations, since range-based measurements provide higher accuracy. For range-free situations, the distance measurements have low accuracy, which directly decreases the localization accuracy. The agents with low localization accuracy do not contribute to improving the localization performance of the whole network but disrupt the whole localization system and may cause localization breakdown.

In this paper, we propose a cooperative localization model for wireless sensor networks in range-free situations. In the distance estimation phase, a multi-hop method is introduced to estimate the node distance. To improve the accuracy of distance estimation, a novel weight allocation way is proposed to offset the deviation from the multi-hop method based on the neighboring anchors. In the localization phase, a hard-decision cooperative method is proposed by analyzing the geometric relationship between agent position and distance. In this way, it is possible to eliminate unreasonable agents and avoid a large deviation by geometric constraints.

The rest of the paper is organized as follows. Section 2 presents the multi-hop distance estimation and the error compensation method. Section 3 studies the cooperative localization method with variational message passing (VMP) rules and derives the geometric constraints and iterative parameters. Numerical results are demonstrated in Section 4, and conclusions are given in Section 5.

## 2. Distance Estimation

In this section, we describe an improved distance estimation method which expands the multi-hop estimation with an exponential distance calibration. The multi-hop estimation regards a localization scenario as a uniformly distributed network, where node density is fully utilized to estimate neighboring nodes’ distance in an adjacent area. The sum of neighboring nodes’ distances in the shortest path is the initial distance of two nodes in the far field and it is divided into even shortest paths and odd shortest paths, manipulated in different ways. Considering the high uncertainty and randomness of the multi-hop method, we propose a calibration method to improve localization accuracy. In the sensor network, a neighboring anchor is very important to locate agents. Here, we evaluate the error range of the multi-hop method and calibrate the distance estimation between two agents because agent to be located may share the same shortest path to other node. The calibration refers to all anchors within the communication range of the agent to be located. According to the estimated distance between anchors and agent, we propose that the anchors influence the agent in an exponential weight form. 

### 2.1. Multi-Hop Method

Consider a wireless network consisting of $M=N_{A}+N_{a}$ nodes, where there are $N_{A}$ anchors and $N_{a}$ agents. We denote the position of the m−th node as $\theta_{m}≜{\left[x_{m},y_{m}\right]}^{T},m\in S\cup A$, where $A=\left\{1,\cdots ,N_{a}\right\}$ stands for the set of the indexes of agents, while $S=\left\{N_{a}+1,\cdots ,M\right\}$ is the set of the indexes of anchors. These sensors are assumed to be uniformly distributed in an area. All anchor and sensor nodes are assumed to have the same range (i.e., transmission capability), denoted by R. Each node is then able to directly communicate with any other node located in the disc having that node as a center and R as a radius, while it communicates in a multi-hop fashion with the nodes located outside it.

**Figure 1:**
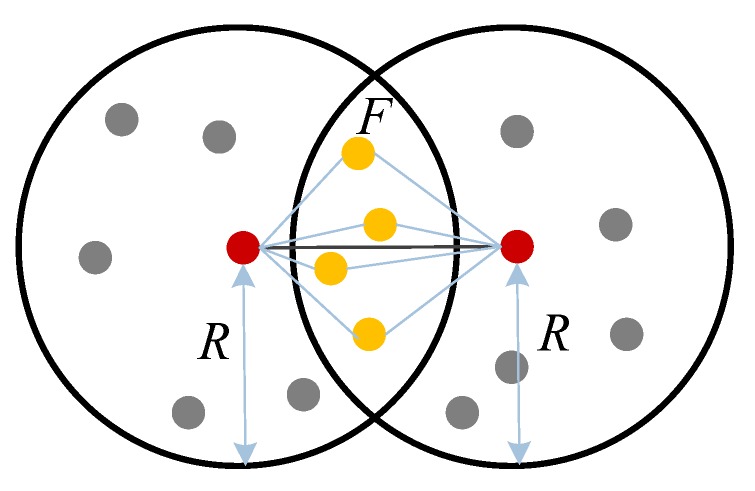
Distance estimation between two nodes in a two-hop range.

We firstly explore the distance estimation between two nodes in a two-hop range. As shown in Figure 1, the red points are the two nodes to be located. They are beyond communication range but can communicate through another one node which is denoted by yellow points. *F* denotes the adjacent area of the two nodes’ communication range. 

As the nodes are assumed to be uniformly distributed, we estimate the area through the node density ρ. Because a wireless sensor network is a kind of ad hoc network, its sensors can be self-organized once all sensors are started in the network. For developed hardware, such as CC2530 or IRIS, each sensor in the network can obtain device information at all times and form an associated device list. This associated device list includes all nodes which are within the communication range of the agent to be located. Once one of these nodes joins or quits the network, the associated device list would be updated. Through the associated device list, sensors can obtain and record their neighboring nodes information. After the ad hoc network is prepared, the agent to be located sends its neighboring information to each node of its own associated device list and receives neighboring information from other sensors. The sensor to be located compares its own neighboring information to that of the neighboring nodes, so that the common nodes are counted. The number of common nodes is N in the adjacent area F. The adjacent area can be expressed as

(1) F=N/ρ

Also, we can express the relationship between adjacent area and node distance as

(2) $$F=\phi \left(d_{ij}\right)=2R^{2}\cos^{-1}\left(\frac{d_{ij}}{2R}\right)-\frac{1}{2}d_{ij}\sqrt{4R^{2}-d_{ij}^{2}}$$

where $d_{ij}$ denotes the distance between node i and j, and i,j=1,⋯,M. 

The node distance is obtained by solving the following equation:

(3) $${\hat{d}}_{ij}=\psi \left(\hat{F}\right)$$

where $\psi \left(\hat{F}\right)$ is the inverse function of $\phi \left(d_{ij}\right)$ and it is also a decreasing function with respect to the adjacent area $\hat{F}$. Because the node distance cannot have a closed-form solution in Equation (3), the Secant method is introduced to solve the root-finding problem. It is based on

(4) $$\tilde{\phi }\left(x\right)=\phi \left(x\right)-\hat{F}$$

The node distance is updated by

(5) $${\hat{d}}_{ij}^{p+1}={\hat{d}}_{ij}^{p}-\tilde{\phi }\left(d_{ij}^{p}\right)\frac{d_{ij}^{p}-d_{ij}^{p-1}}{\phi \left(d_{ij}^{p}\right)-\phi \left(d_{ij}^{p-1}\right)}$$

where p denotes the number of iterations. When the condition $p=p^{\max}=\inf_{p}\left\{{\hat{d}}_{ij}^{p}={\hat{d}}_{ij}^{p+s},\forall s\in N^{*}\right\}$ is met, the iteration stops. To obtain ${\hat{d}}_{ij}^{p}={\hat{d}}_{ij}^{p^{\max}}$, we firstly set ${\hat{d}}_{ij}^{0}=R,{\hat{d}}_{ij}^{0}=2R.$

The distance estimation above is for two nodes in a two-hop range. When the distance of two nodes is beyond the two-hop range, it can be estimated by the shortest path. If the hops in the shortest path are even, the distance is

(6) $${\hat{d}}_{ij}=\sum_{l=1}^{n_{h}/2}\Psi \left(\frac{N_{l}}{\rho }\right)$$

**Figure 2:**
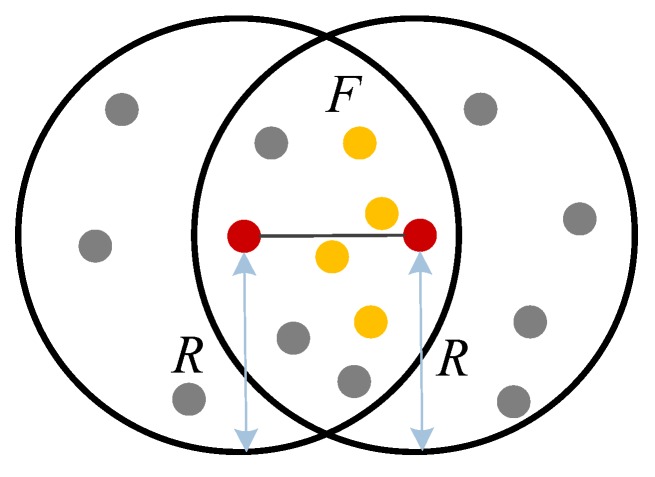
Distance estimation between two nodes within communication range.

If the hops in the shortest path are odd, the distance of the last hop needs to be further estimated as shown in Figure 2.

In the same way, Equation (2) provides a solution for the distance estimation for a single hop. The adjacent area is estimated by the common node in their communication ranges. When the shortest path is odd, the node distance is

(7) $${\hat{d}}_{ij}=\sum_{l=1}^{\left(n_{h}-1\right)/2}\Psi \left(\frac{N_{l}}{\rho }\right)+d_{av}^{Last}$$

### 2.2. Distance Calibration

Although the nodes are assumed to be uniformly distributed in the area, homogeneity is not possible in practice. This assumption leads to the inaccurate estimation of the area, which decreases the accuracy of distance measurement. Also, the nonlinear shortest path cannot reflect the linear distance between two nodes, and considering it leads to an error in distance estimation. To alleviate the effect of these assumptions, we extract path information from the neighboring anchors. The relevance of agent and neighboring anchors in non-cooperative networks has been investigated in [22]. For a cooperative network, we should find a better way to calibrate the distance error.

**Figure 3:**
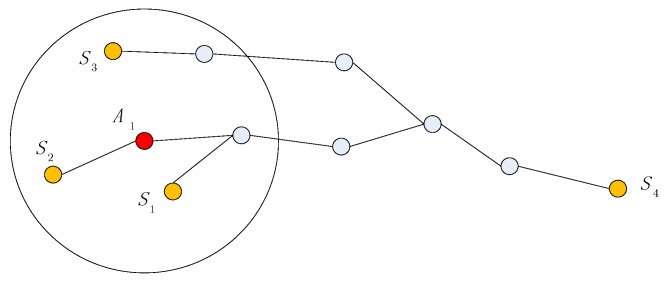
Shortest path between agent and anchor.

Distance calibration includes two scenarios: distance between agent and anchor, and distance between agent and agent. For agent $A_{1}$ and anchor $S_{4}$, the shortest path is shown in Figure 3. Points $S_{1},S_{2},S_{3}$ are the neighboring anchors within communication range of agent $A_{1}$. Because agent $A_{1}$ and its neighboring anchors $S_{1},S_{2},S_{3}$ may share the same path, the distance between anchors could provide reference for distance calibration.

On this basis, we propose the distance calibration formula

(8) $$\Theta_{A_{1}S_{4}}=\sum_{i=1}^{\kappa }\lambda_{i}(d_{S_{i}S_{4}}-{\hat{d}}_{S_{i}S_{4}})$$

where $\Theta_{A_{1}S_{4}}$ is the calibration value of agent $A_{1}$ and anchor $S_{4}$, $d_{S_{i}S_{4}}$ is the true distance between anchors $S_{i}S_{4}$, ${\hat{d}}_{S_{i}S_{4}}$ is the distance estimated by the multi-hop method in Section 2.1, $\lambda_{i}$ is the weight of each distance, κ is the number of selected anchors, which depends on two factors, one of which is the neighboring anchors number of agent $A_{1}$. If this number is small, all the neighboring anchors contribute to the distance calibration. If the number is large, only part of them contribute to the distance calibration. The larger the number of neighboring anchors, the larger their over-calibration on distance estimation. Another factor affecting the selection of anchors is the distance between agent $A_{1}$ and neighboring anchor. Usually, the anchor at a lower distance more probably shares the same shortest path. On this basis, the number κ as well as the weight $\lambda_{i}$ are determined. To characterize the distance information better, we propose an exponential weight allocation formula

(9) $$\lambda_{i}=e^{-{\overset{⌢}{d}}_{A_{1}S_{i}}}/\sum_{i=1}^{\kappa }e^{-{\hat{d}}_{A_{1}S_{i}}}$$

After calibration, the distance between agent and anchor is

(10) $${\tilde{d}}_{A_{1}S_{4}}={\hat{d}}_{A_{1}S_{4}}+\eta_{A_{1}S_{4}}\Theta_{A_{1}S_{4}}$$

where $\eta_{A_{1}S_{4}}$ is the calibration coefficient, which is designed to avoid over-calibration. Usually, the calibration coefficient is set to $0<\eta_{A_{1}S_{4}}<1$.

**Figure 4:**
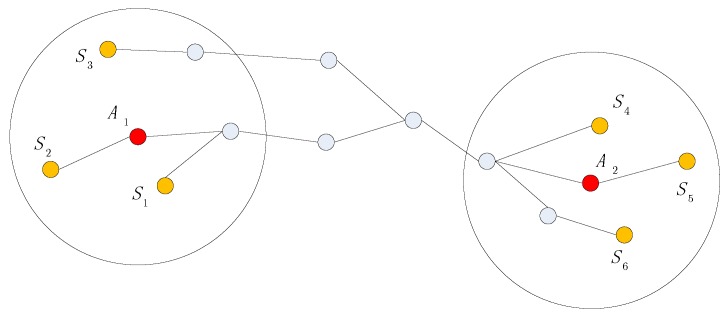
Shortest path between agents.

For agent $A_{1}$ and agent $A_{2}$, the shortest path is shown in Figure 4. Points $S_{1},S_{2},S_{3}$ are the neighboring anchors within communication range of agent $A_{1}$. Points $S_{4},S_{5},S_{6}$ are the neighboring anchors within communication range of agent $A_{2}$.

In the same way, the distance calibration formula for agents is

(11) $$\Theta_{A_{1}A_{2}}=\sum_{i=1}^{\kappa_{i}}\sum_{j=1}^{\kappa_{j}}\lambda_{ij}(d_{S_{i}S_{j}}-{\hat{d}}_{S_{i}S_{j}})$$

where $\kappa_{i}$ denotes the selected anchors around agent $A_{1}$, and $\kappa_{j}$ denotes the selected anchors around agent $A_{2}$. Their weights are also allocated by distances. The exponential form of weight is

(12) $$\lambda_{ij}=\left(e^{-{\hat{d}}_{A_{1}S_{i}}}+e^{-{\overset{⌢}{d}}_{A_{2}S_{j}}}\right)/\sum_{i=1}^{\kappa_{i}}\sum_{j=1}^{\kappa_{j}}\left(e^{-d_{A_{1}S_{i}}}+e^{-d_{A_{2}S_{j}}}\right)$$

After calibration, the distance between the agents is

(13) $${\tilde{d}}_{A_{1}A_{2}}={\hat{d}}_{A_{1}A_{2}}+\eta_{A_{1}A_{2}}\Theta_{A_{1}A_{2}}$$

where $\eta_{A_{1}A_{2}}$ is the calibration coefficient, which is usually $0<\eta_{A_{1}A_{2}}<1$.

## 3. Cooperative Localization

For range-free localization, a large deviation of distance estimation and position estimation may often happen, due to scarce information and difficult communication environment. These unwelcome estimations are called outliers. Especially in cooperative localization, outliers may lead to the failure of whole network. To alleviate the effect of the outliers, we propose a cooperative localization method with outlier constraints. Outlier constraints are divided into distance constraints and position constraints, which both rely on neighboring anchors. The estimated distance and position will not exceed the communication range of neighboring anchors for both distance constraints and position constraints. These constraints are represented by the parameters α and β, which condition the cooperative method. The cooperative method introduces message passing and updates the estimated position through the variational message passing rule. The final localization result depends on confidence of the agent to be located.

### 3.1. Outlier Constraints

For range-based localization, the estimated distances are relatively accurate, so that the localization of agents is quite precise. For range-free localization, in our paper, the accuracy fails to meet the requirement of cooperative localization for both node distance and node position. If the outlier distance and position are regarded as key information of a virtual anchor, this will cause a large derivation in the subsequent iteration, leading, finally, to disruption of the whole network and localization failure. To avoid this problem, we propose the outlier constraint method, which includes position constraint and distance constraint.

**Figure 5:**
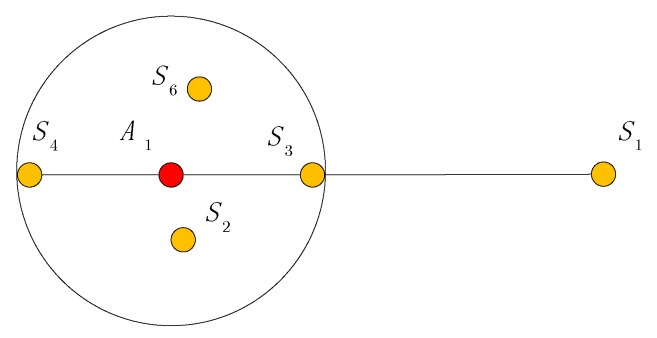
Outlier constraint of agent and anchor’s distance.

**Figure 6:**
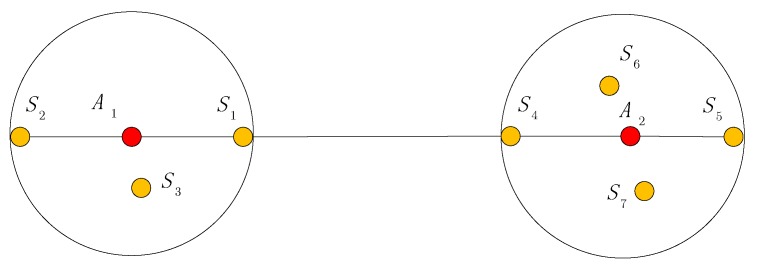
Outlier constraint of agents’ distance.

It is inevitable to have low accuracy of distance estimation with the multi-hop method. The outlier distance has a negative effect on cooperative localization, so it is better to exclude the outlier distances. The outlier distance is excluded by constraints from anchors, as shown in Figure 5 and Figure 6. Figure 5 shows the distance between agent and anchor. Figure 6 shows the distance between agents. For the distance between agent and anchor, the outlier constraint is

(14) $$d_{SS_{1}}-R\leq {\tilde{d}}_{A_{1}S}\leq d_{SS_{1}}+R$$

where ${\tilde{d}}_{A_{1}S}$ is the estimated distance according to Section 2, S denotes the neighboring anchors within communication range of $A_{1}$, $d_{SS_{1}}$ denotes the true distance between anchor $S_{1}$ and any anchor in S. 

Hence, we propose a constraint function as

(15) $$\alpha_{A_{1}S}\left({\tilde{d}}_{A_{1}S}\right)=\left\{ \begin{array}{l} 1,d_{SS_{1}}-R\leq {\tilde{d}}_{A_{1}S}\leq d_{SS_{1}}+R\\ 0,\mathrm{others} \end{array}\right.$$

For the distance between agents, the outlier constraint is

(16) $$d_{SS’}-2R\leq {\tilde{d}}_{A_{1}A_{2}}\leq d_{SS’}+2R$$

where ${\tilde{d}}_{A_{1}A_{2}}$ is the estimated distance according to Section 2, S denotes the neighboring anchors within communication range of $A_{1}$, S’ denotes the neighboring anchors within communication range of $A_{2}$, $d_{SS’}$ denotes the real distance between any anchor in S and any anchor in S’. 

Hence, we propose the constraint function

(17) $$\alpha_{A_{1}A_{2}}\left({\tilde{d}}_{A_{1}A_{2}}\right)=\left\{ \begin{array}{l} 1,d_{SS’}-2R\leq {\tilde{d}}_{A_{1}A_{2}}\leq d_{SS’}+2R\\ 0,\mathrm{others} \end{array}\right.$$

In a cooperative network, the outlier position of the agent also has a negative effect on cooperative localization. It is better to exclude these outlier positions through neighboring anchors. In the network, agents can communicate with all nodes that are in their communication range. Therefore, distance between the agent and its neighboring anchor is lower than the communication range:

(18) $${\left\|{\hat{\theta }}_{A_{1}}-\theta_{S}\right\|}_{2}\leq R$$

where ${\hat{\theta }}_{A_{1}}$ is the estimated position of agent $A_{1}$, S denotes the neighboring anchors within communication range of $A_{1}$, $\theta_{S}$ denotes position of any anchor in S.

Hence, we propose the constraint function

(19) $$\beta_{A_{1}}\left({\hat{\theta }}_{A_{1}}\right)=\left\{ \begin{array}{l} 1,{\left\|{\hat{\theta }}_{A_{1}}-\theta_{S}\right\|}_{2}\leq R\\ 0,\mathrm{others} \end{array}\right.$$

where ‖⋅‖ is the Euclidean norm of estimated position ${\hat{\theta }}_{A_{1}}$ and true position $\theta_{S}$.

### 3.2. Cooperative Localization

Thanks to the locally factorized structure of the joint likelihood function, the cooperative localization problem can be addressed under the framework of factor graph. Inference and estimation tasks are typically carried out on a factor graph by message passing between variable nodes and factor nodes. Existing methods do not consider the outlier variable nodes and factor, which indicates that cooperative localization is not suitable for a range-free situation. In the cooperative phase, we explore a hard decision-based cooperative way for a range-free situation applying outlier constraints. 

We assume that the sensor node i acquires a noisy measurement ${\tilde{d}}_{ij}$ and that i,j∈S∪A, which is the estimated distance from the sensor node j (j can be either an agent or an anchor). Then, we have

(20) $${\tilde{d}}_{ij}=\left\|\theta_{i}-\theta_{j}\right\|+e_{ij}$$

where $e_{ij}$ is the measurement noise. Without loss of generality, we assume that measurement noise obeys a Gaussian distribution, so $e_{ij}∼N\left(e_{ij};0,\sigma_{ij}^{2}\right)$; $\sigma_{ij}$ is the standard deviation. The probability density function of node distance is

(21) $$p\left({\tilde{d}}_{ij}|\theta_{i},\theta_{j}\right)=\frac{1}{\sqrt{2\pi }\sigma_{ij}}\exp\left\{-\frac{{\left({\tilde{d}}_{ij}-\left\|\theta_{i}-\theta_{j}\right\|\right)}^{2}}{2\sigma_{ij}^{2}}\right\}$$

We define $f_{ij}≜p\left({\tilde{d}}_{ij}|\theta_{i},\theta_{j}\right)$ and assume that the relative positions are conditionally independent and only depend on the two nodes involved:

(22) $$p\left(D|\theta \right)=\prod_{i\in S\cup A}\prod_{j\in S\cup A}p\left({\tilde{d}}_{ij}|\theta_{i},\theta_{j}\right)$$

where θ is defined as the position set $\theta ≜\left\{\theta_{i}:i\in S\cup A\right\}$ of all nodes, and D is defined as the distance set $D≜\left\{{\tilde{d}}_{ij}:i\in S\cup A,j\in S\cup A\right\}$ of all nodes.

Among various message-passing algorithms, belief propagation (BP, also known as sum-product algorithm, SPA) and VMP are the most widely applied ones. Compared with BP, VMP can often lead to relatively simple message-passing rules, which roots from the fact that VMP enforces stronger constraint on the form of trail distributions. Considering the effect of the outliers, we propose a hard decision-based cooperative method and define a VMP rule with outlier constraints as

(23) $$m_{f_{ia}\to \theta_{i}}\left(\theta_{i}\right)=\exp\left(\int \alpha_{ia}\left({\tilde{d}}_{ia}\right)b\left(\theta_{a}\right)\ln p\left({\tilde{d}}_{ia}|\theta_{i},\theta_{a}\right)d\theta_{a}\right)$$

(24) $$m_{f_{il}\to \theta_{i}}\left(\theta_{i}\right)=\exp\left(\int \alpha_{il}\left({\tilde{d}}_{il}\right)\beta_{i}\left({\hat{\theta }}_{i}\right)b\left(\theta_{l}\right)\ln p\left({\tilde{d}}_{il}|\theta_{i},\theta_{l}\right)d\theta_{l}\right)$$

where $m_{f_{ia}\to \theta_{i}}\left(\theta_{i}\right),m_{f_{il}\to \theta_{i}}\left(\theta_{i}\right)$ are the messages from anchor factor $f_{ia}$ and agent factor $f_{il}$, $b\left(\theta_{a}\right),b\left(\theta_{l}\right)$ are the confidence result in the last iteration, $\alpha_{ia}\left({\tilde{d}}_{ia}\right),\alpha_{il}\left({\tilde{d}}_{il}\right)$ are the outlier constraints of the estimated distance, $\beta_{i}\left({\hat{\theta }}_{i}\right)$ is the outlier constraint of the estimated position. Their definition was presented in Section 3.1. When the estimated distance or position is identified as outlier, the messages are invalid. This ensures that the outliers do not disrupt the whole network. 

An approximation of the posterior distribution is

(25) $$b\left(\theta_{i}\right)≜\frac{1}{Z}\prod_{a\in S}m_{f_{ia}-\theta_{i}}\left(\theta_{i}\right)\prod_{l\in A}m_{f_{il}-\theta_{i}}\left(\theta_{i}\right)$$

where Z is a normalization coefficient.

After some manipulation, the confidence of the agent position is

(26) $$b\left(\theta_{i}\right)\propto \exp\{\sum_{a\in S}\alpha_{ia}\left({\tilde{d}}_{ia}\right)\cdot g_{ia}\left(\theta_{i}\right)+\sum_{l\in A}\alpha_{il}\left({\tilde{d}}_{il}\right)\cdot \beta_{i}\left({\hat{\theta }}_{i}\right)\cdot g_{il}\left(\theta_{i}\right)\}$$

where

(27) $$g_{ia}\left(\theta_{i}\right)≜\frac{{\tilde{d}}_{ia}}{{\left(\sigma_{ia}\right)}^{2}}\left\|\theta_{a}-\theta_{i}\right\|-\frac{1}{2{\left(\sigma_{ia}\right)}^{2}}{\left\|\theta_{a}-\theta_{i}\right\|}^{2}$$

(28) $$g_{il}\left(\theta_{i}\right)≜\int \hat{b}\left(\theta_{l}\right)\left(\frac{{\tilde{d}}_{il}}{{\left(\sigma_{il}\right)}^{2}}\left\|\theta_{l}-\theta_{i}\right\|-\frac{1}{2{\left(\sigma_{il}\right)}^{2}}{\left\|\theta_{l}-\theta_{i}\right\|}^{2}\right)$$

We expand the Euclidean norms in the equations with second-order Taylor expansion. The Euclidean norms $F_{ia}\left(\theta_{i}\right)≜\left\|\theta_{a}-\theta_{i}\right\|$ and $F_{il}\left(\theta_{i},\theta_{l}\right)=\left\|\theta_{l}-\theta_{i}\right\|$ are respectively expanded around ${\hat{\theta }}_{i}^{*}$ and $\left({\hat{\theta }}_{i}^{*},{\hat{\theta }}_{l}^{*}\right)$. Then, equation (26) is transformed as

(29) $$\hat{b}\left(\theta_{i}\right)\propto \exp\left\{-\frac{1}{2}{\left(\theta_{i}\right)}^{T}{\left({\hat{V}}_{i}\right)}^{-1}\theta_{i}+{\left(\theta_{i}\right)}^{T}{\left({\hat{V}}_{i}\right)}^{-1}{\hat{\theta }}_{i}\right\}$$

where ${\hat{V}}_{i}$ is defined as variance, and ${\hat{\theta }}_{i}$ is the expectation of the agent position. They are iteratively calculated by

(30) $${\hat{V}}_{i}=\{\sum_{a\in S}\alpha_{ia}\left({\tilde{d}}_{ia}\right)\cdot \left(\frac{1}{{\left(\sigma_{ia}\right)}^{2}}I-\frac{{\tilde{d}}_{ia}}{{\left(\sigma_{ia}\right)}^{2}}\nabla_{F_{ia}}^{2}\right){+\sum_{l\in A}\alpha_{il}\left({\tilde{d}}_{il}\right)\cdot \beta_{i}\left({\hat{\theta }}_{i}\right)\cdot \left(\frac{1}{{\left(\sigma_{il}\right)}^{2}}I-\frac{{\tilde{d}}_{il}}{{\left(\sigma_{il}\right)}^{2}}H_{F_{il}}\right)\}}^{-1}$$

(31) $$\begin{array}{ll} {\hat{\theta }}_{i}= & {\hat{V}}_{i}(\sum_{a\in S}\alpha_{ia}\left({\tilde{d}}_{ia}\right)\cdot \left(\frac{1}{{\left(\sigma_{ia}\right)}^{2}}\theta_{a}+\frac{{\tilde{d}}_{ia}}{{\left(\sigma_{ia}\right)}^{2}}\left(\nabla_{F_{ia}}-\nabla_{F_{ia}}^{2}{\tilde{\theta }}_{i}\right)\right)\\ & +\sum_{l\in A}\alpha_{il}\left({\tilde{d}}_{il}\right)\cdot \beta_{i}\left({\tilde{\theta }}_{i}\right)\cdot \left(\frac{1}{{\left(\sigma_{il}\right)}^{2}}{\tilde{\theta }}_{l}+\frac{{\tilde{d}}_{il}}{{\left(\sigma_{il}\right)}^{2}}\left(\nabla_{F_{il}}-H_{F_{il}}{\tilde{\theta }}_{i}\right)\right)) \end{array}$$

where $\tilde{\theta }$ denotes the estimated position of the last iteration, $\nabla_{F_{ia}},\nabla_{F_{ia}}^{2}$ are the first-order and second-order gradient of $F_{ia}\left(\theta_{i}\right)$ at ${\hat{\theta }}_{i}^{*}$, $\nabla_{F_{il}},H_{F_{il}}$ is the first-order partial derivative and Hessian matrix with respect to $F_{il}\left(\theta_{i},\theta_{l}\right)$ at $\left({\hat{\theta }}_{i}^{*},{\hat{\theta }}_{l}^{*}\right)$.

The network operates in a cooperative way through Equations (30) and (31) until pre-defined iteration count. The final values of ${\hat{V}}_{i}$ and ${\hat{\theta }}_{i}$ are the localization results.

## 4. Results and Discussion

Node locations and populations play a key role in determining the performance of a localization network due to their stochastic natures which lead to changes of network topology. In this section, we consider the general behaviors of a stochastic localization network where anchors and/or agents are randomly distributed in the two-dimensional space. On this basis, the performance of the proposed algorithm is evaluated through numerical simulations.

### 4.1. Localization Performance

**Figure 7:**
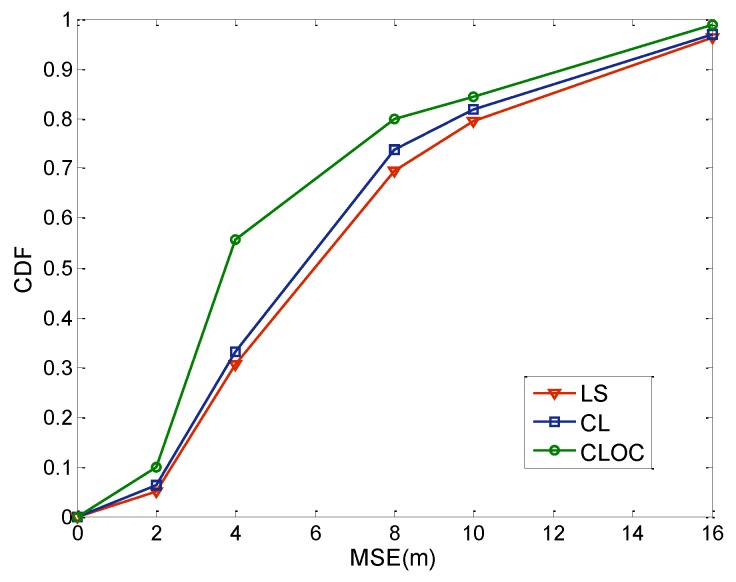
Comparison of the localization results. LS: least-squares method, CL: cooperative localization without outlier constraints, CLOC: cooperative localization with outlier constraints.

We first investigate the localization performances of the proposed method and traditional method in a network with 40 anchors and 160 agents. The communication radius is assumed to be 30 m, and the distribution area is 100 m × 100 m. The node density is 0.02/m^2^. The number of iterations for algorithms is set to 10. The parameters η in Equations (10) and (13) are 0.5. Standard deviations of the estimated positions are $\sigma_{ia}\;=\;2$ for anchors and $\sigma_{il}\;=\;4$ for agents. For comparison, we select the least-squares (LS) method, cooperative localization without outlier constraints (CL), and cooperative localization with outlier constraints (CLOC). The localization results are depicted by cumulative distribution functions (CDFs), as shown in Figure 7. 

As shown in Figure 7, CLOC outperforms LS and CL in accuracy. The mean-square error (MSE) of CLOC is 5.225 m, while the MSE of LS and CL are, respectively, 7.665 and 6.92 m. Compared with LS, CLOC improves the accuracy by about 30%. For CL and CLOC, the localization results are almost same when the localization error is small, while CLOC outperforms CL when the localization error is large. The reason for the better performance of CLOC is that the outlier constraints limited the possible distribution of the estimated position. If outliers are considered, outlier constraints will improve the localization of the whole network. Therefore, most localization errors in the CLOC method are less than 4 m. Actually, CL and CLOC are both cooperative methods. LS is a non-cooperative method. In Figure 7, cooperative methods show better performance than non-cooperative methods. Because outliers may lead to the wrong direction of iterative adjustment, the advantage of the cooperative method is not obvious. As the outliers are considered in the CLOC method, the localization accuracy is largely improved.

### 4.2. Performance of Distance Estimation

Distance estimation is a key step in the localization of a WSN. In this subsection, we investigate the performance of distance estimation and the effect of the calibration coefficient. We consider a network where 40 anchors and 160 agents are uniformly distributed in a 100 m × 100 m area. The node density is 0.02/m^2^, and the communication radius is 30 m. The calibration coefficient varies from 0 to 1. When the calibration coefficient is 0, the distance calibration does not affect the distance estimation. We compare the distance estimation results in different scenarios, as shown below. 

**Figure 8:**
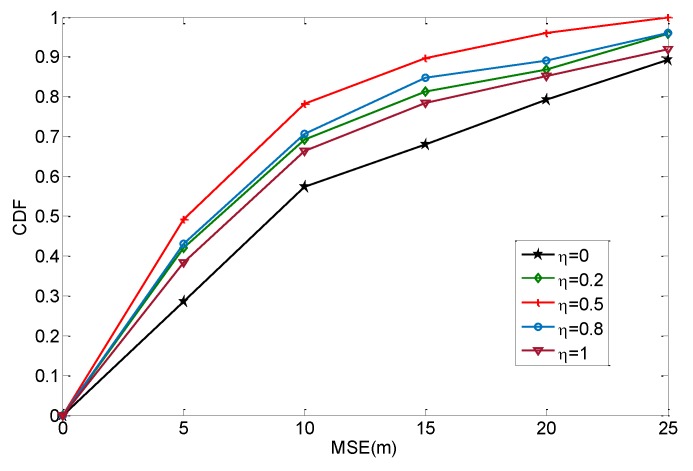
Comparison of the distance results.

As shown in Figure 8, the estimated results present different performances with different calibration coefficients. The performance is the worst when the calibration coefficient is 0. This indicates that the traditional multi-hop method does not fully utilize the information from anchors, while its performance can be improved by neighboring anchors. Other curves show the results after distance calibration. Actually, the calibration coefficients have different effects on distance estimation. In Figure 8, the estimation results are better when the calibration coefficient is 0.5, while they are worse when the calibration coefficient is 1. This indicates that over-calibration is not always good for distance estimation. Although the error from anchors can provide a reference, it is not completely valid for the agent. If the calibration value is totally introduced in the distance estimation, it causes over-calibration that may increase the error in an opposite manner. In the scenario of this subsection, the most suitable value for the calibration coefficient is 0.5.

To further explore how to set the calibration coefficient, we design several simulations conducted in different scenarios. The first scenario (Scenario I) considers a network where 80 anchors and 320 agents are uniformly distributed in a 100 m × 100 m area. The node density is 0.04/m, and the communication radius is 30 m. Scenario II considers a network where 20 anchors and 180 agents are uniformly distributed in a 100 m × 100 m area. The node density is 0.02/m^2^, and the communication radius is 30 m. Scenario III considers a network where 40 anchors and 160 agents are uniformly distributed in a 100m × 100m area. The node density is 0.02/m^2^, and the communication radius is 20 m. Scenario IV considers a network where 40 anchors and 160 agents are uniformly distributed in a 100 m × 100 m area. The node density is 0.02/m^2^, and the communication radius is 50 m. For these scenarios, we also select five calibration coefficient values to compare the localization performance. These values are 0, 0.2, 0.5, 0.8, and 1. The values with the best localization accuracy are shown in Table 1.

Table 1 shows that the optimum calibration coefficient is 0.2 for Scenario IV, whereas it is 0.5 for all the other scenarios. This indicates in most cases, the localization performance is better when the calibration coefficient is 0.5. The reason for a different optimum value in Scenario IV is the large ratio between communication radius and distribution area. When the communication radius is 50 m and the side of distribution area is 100 m, most sensors have at most three hops to reach other nodes in the shortest path. Therefore, the theory of sharing the same path can rarely be used in the localization method. This leads to a small calibration coefficient in the distance calibration phase. In practical engineering, the calibration coefficient is usually set as 0.5, except in special cases similar to Scenario IV.

### 4.3. Effect of Network Distribution Setting

For range-free methods, network distribution parameters, such as node density, anchor proportion, and communication radius, have a big influence on the localization results. In this subsection, we investigate the effect of these network distribution parameters. 

**Figure 9:**
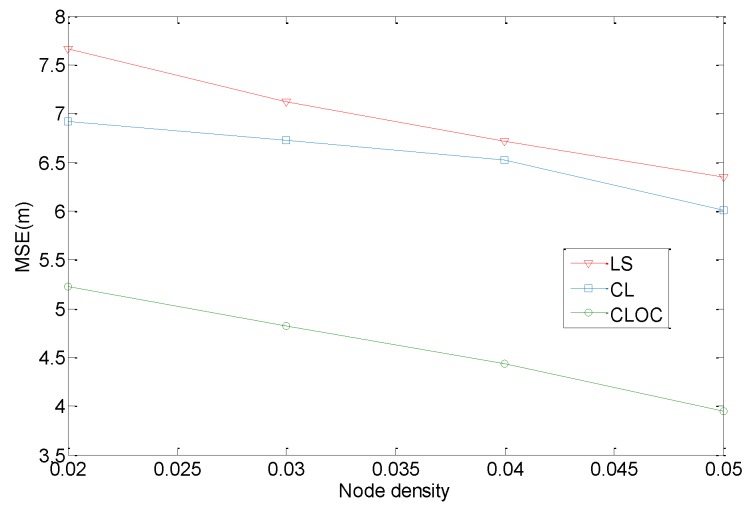
Comparison of the localization performance with respect to node density.

Node density is firstly investigated, with these nodes uniformly distributed in a 100 m × 100 m area. The communication radius is 20 m, and the anchor proportion is 20%. The number of iterations for algorithms is set to 10. The parameters η in Equations (10) and (13) are 0.5. The standard deviations of the estimated positions are, respectively, $\sigma_{ia}\;=\;2$ for anchors and $\sigma_{il}\;=\;4$ for agents. The total node number varies from 200 to 500, so the node density varies from 0.02/m^2^ to 0.05/m^2^. The localization results are shown in Figure 9.

Figure 9 shows the comparison of the performance of LS, CL, and CLOC. Due to the outliers, the accuracy of CL without outlier constraints is close to that of with LS, and both CL and LS underperform compared with CLOC. The accuracy of the three methods increases with increasing node density. There are two reasons for their good performance. One is that a high node density helps estimate the distance, because the nodes are more uniform, and the area range is easier to differentiate. Another reason is that more anchors and agents can provide more references. Therefore, CLOC outperforms in localization accuracy, and a high node density contributes to the improvement.

**Figure 10:**
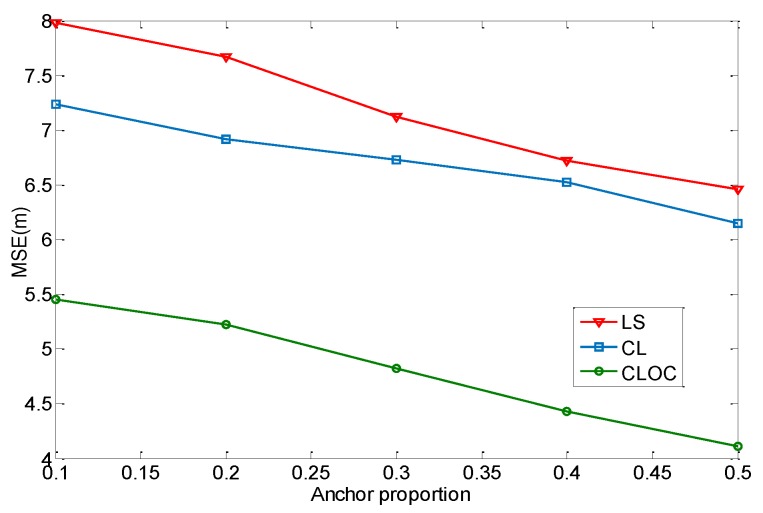
Comparison of the localization performance with respect to anchor proportion.

Anchor proportion also has an effect on the localization performance. To investigate this effect, 200 nodes are uniformly distributed in a 100 m × 100 m area. The node density is 0.02/m^2^, and the communication radius is 20 m. The number of iterations for algorithms is set to 10. The parameters η in Equations (10) and (13) are 0.5. The standard deviations of the estimated positions are $\sigma_{ia}\;=\;2$ for anchors and $\sigma_{il}\;=\;4$ for agents. The anchor proportion varies from 20% to 50%. The localization results are shown in Figure 10.

As shown in Figure 10, CLOC outperforms in accuracy for all scenarios compared with LS and CL. This indicates a good improvement produced by the proposed method, without the addition of excessive computation. Meanwhile, the accuracy of the three methods increases with an increasing anchor proportion. This is because the anchors’ positions are more accurately determined, and the anchors are the reference to localize the agents. Therefore, a high anchor proportion can effectively decrease the localization error.

**Figure 11:**
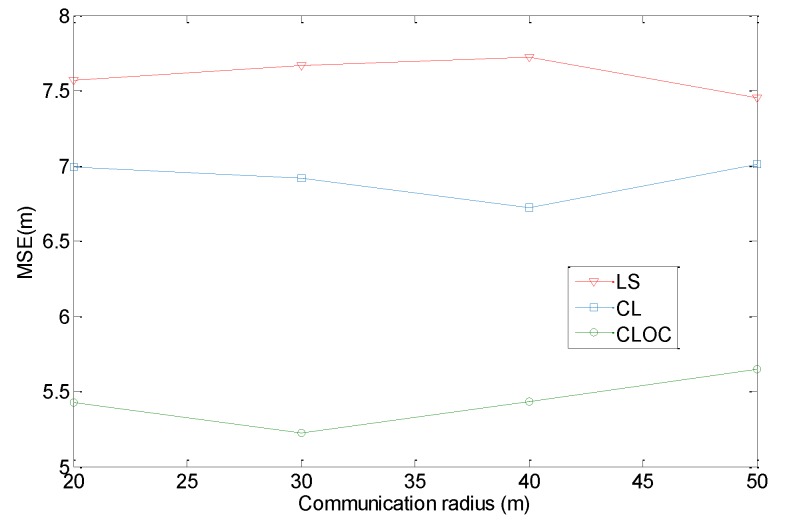
Comparison of the localization performance with respect to the communication radius.

We also investigate the effect of the communication radius on the localization performance. Here, 160 agents and 40 anchors are uniformly distributed in a 100 m × 100 m area. The node density is 0.02/m^2^. The number of iterations for algorithms is set to 10. The parameters η in Equations (10) and (13) are 0.5. The standard deviations of the estimated positions are $\sigma_{ia}\;=\;2$ for anchors and $\sigma_{il}\;=\;4$ for agents. The communication radius varies from 20 to 50 m. The localization results are shown in Figure 11.

Figure 11 shows that CLOC outperforms for any communication radius compared with LS and CL. However, the localization performance has no relationship with the communication radius. The trend of localization accuracy does not change with respect to the communication radius. The reasons for this are as following. Although a larger communication radius can consist of more nodes, the number of nodes to differentiate the area of distribution is not higher. In the distance estimation phase, a larger communication radius does not improve the distance accuracy. In the localization phase, more agents are included to realize cooperative localization when the communication increases. However, these agents are unable to provide a precise reference, while the availability of outlier constraints is weakened. Therefore, the communication radius has little effect on the localization results.

**Figure 12:**
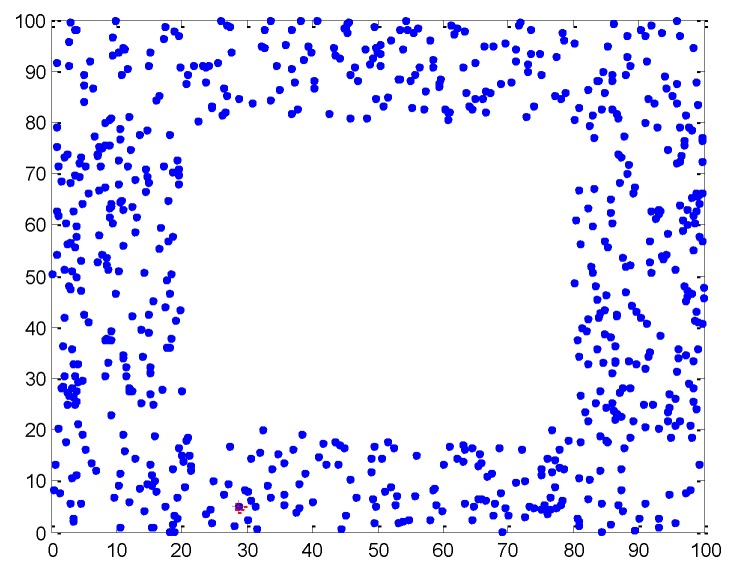
Non-homogeneous distributions with holes or obstacles.

**Figure 13:**
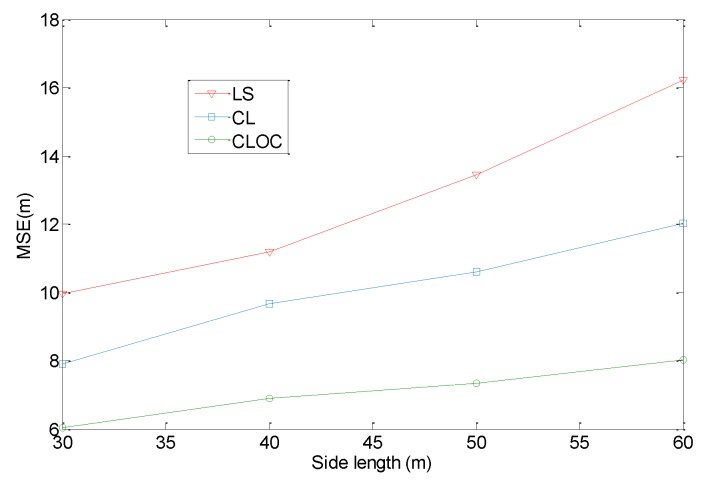
Comparison of the localization performance with respect to the side length of holes or obstacles.

The proposed method is verified in a simulation environment, using a simple model of wireless sensor network, which may not reflect real-world scenarios. To simulate a practical network, additional experiments are performed to demonstrate the advantages of the method for non-homogeneous distributions of nodes. We consider a network distribution with holes or obstacles, as shown in Figure 12. In the network, 160 agents and 40 anchors are uniformly distributed in a 100 m × 100 m area, excluding holes and obstacles. The communication radius is 30 m. The number of iterations for algorithms is set to 10. The parameters η in Equations (10) and (13) are 0.5. The standard deviations of the estimated positions are $\sigma_{ia}\;=\;2$ for anchors and $\sigma_{il}\;=\;4$ for agents. The hole or obstacle is assumed to be a square with its side varying from 30 to 60 m. the localization results are shown in Figure 13.

Figure 13 shows the localization results with respect to the side length of holes or obstacles. Compared with a homogeneous network, LS, CL, and CLOC decrease the localization accuracy. As the side length of holes or obstacles increases, the localization error increases. Therefore, in a non-homogeneous situation, the localization performance decreases. However, the effect of a non-homogeneous distribution is different for LS, CL, and CLOC. As shown in Figure 13, the effect on LS is the largest. Because holes or obstacles directly cause a large distance estimation error, and no measures have been taken to alleviate it, LS localization is seriously affected. Compared with LS, CL and CLOC are both calibrated in the distance estimation phase. Although the shortest path is nonlinear as a result of holes or obstacles, it is eliminated by neighboring anchors’ calibration. In this condition, CL and CLOC are less sensitive to variations of the side length of holes or obstacles compared with LS. Because outlier constraints are used to improve cooperative localization, CLOC outperforms in localization accuracy compared with CL.

### 4.4. Communication Overhead

For localization technology in large-scale WSNs, the method is expected to be highly efficient, and thus it requires a low communication overhead. In this section, CLOC is compared with LS and CL with respect to the communication overhead. The communication overhead of the three methods is shown in Table 2.

Table 2 shows the approximate packages for the localization, where *N_A_* denotes the number of anchors, *N_a_* denotes the number of agents, and *T* denotes the iteration. Among the three methods, LS has the lightest communication load. CL and CLOC have the same communication load. The difference between LS and the cooperative methods is regards the iterated localization process and depends on the iteration counts. For CLOC, the constraints process does not increase the communication load. Considering the improvement of localization accuracy, the communication overhead of the proposed method is acceptable. 

## 5. Conclusions

In this paper, we established a hard decision-based cooperative localization method for wireless sensor networks. In the distance estimation phase, a novel calibration formula was defined to improve the accuracy of the multi-hop method, where the exponential form of weight can better characterize the distance relationship among nodes. In the cooperative localization phase, a hard decision-based method with outlier constraints was proposed to solve the problem for range-free estimation. It effectively avoids large errors in node localization. Simulations were conducted to investigate localization performance, effect of algorithm parameters, and effect of network distribution. In these scenarios, the proposed method showed a good performance for localization accuracy and communication overhead, which promotes the development of low-cost WSN. Further study will focus on the localization capacity in complex environments.

## Figures and Tables

**Table 1:** Optimum calibration coefficients in different scenario.

Scenario	Optimum Calibration Coefficient
Scenario I	0.5
Scenario II	0.5
Scenario III	0.5
Scenario IV	0.2

**Table 2:** Comparison of the three method with respect to communication overhead.

Algorithm	Package
LS	*O*(*N_A_N_a_*)
CL	*O*((*N_A_+T*)*N_a_*)
CLOC	*O*((*N_A_+T*)*N_a_*)
